# The acidified drinking water-induced changes in the behavior and gut microbiota of wild-type mice depend on the acidification mode

**DOI:** 10.1038/s41598-021-82570-0

**Published:** 2021-02-03

**Authors:** Brandon Whipple, Jennifer Agar, Jing Zhao, David A. Pearce, Attila D. Kovács

**Affiliations:** 1 Animal Resource Center, Sanford Research, Sioux Falls, SD 57104 USA; 2 Population Health Group, Sanford Research, Sioux Falls, SD 57104 USA; 3 Pediatrics and Rare Diseases Group, Sanford Research, 2301 E. 60th Street N., Sioux Falls, SD 57014 USA; 4 Department of Internal Medicine, Sanford School of Medicine, University of South Dakota, Sioux Falls, SD 57105 USA; 5 Department of Pediatrics, Sanford School of Medicine, University of South Dakota, Sioux Falls, SD 57105 USA; 6 Present Address: Center for Biostatistics, Ohio State University, Columbus, OH 43210 USA

**Keywords:** Microbiology, Physiology

## Abstract

Acidification of drinking water to a pH between 2.5 and 3.0 is widely used to prevent the spread of bacterial diseases in animal colonies. Besides hydrochloric acid (HCl), sulfuric acid (H_2_SO_4_) is also used to acidify drinking water. Here we examined the effects of H_2_SO_4_-acidified drinking water (pH = 2.8) received from weaning (postnatal day 21) on the behavior and gut microflora of 129S6/SvEv mice, a mouse strain commonly used in transgenic studies. In contrast to HCl-acidified water, H_2_SO_4_-acidified water only temporarily impaired the pole-descending ability of mice (at 3 months of age), and did not change the performance in an accelerating rotarod test. As compared to 129S6/SvEv mice receiving non-acidified or HCl-acidified drinking water, the gut microbiota of 129S6/SvEv mice on H_2_SO_4_-acidified water displayed significant alterations at every taxonomic level especially at 6 months of age. Our results demonstrate that the effects of acidified drinking water on the behavior and gut microbiota of 129S6/SvEv mice depends on the acid used for acidification. To shed some light on how acidified drinking water affects the physiology of 129S6/SvEv mice, we analyzed the serum and fecal metabolomes and found remarkable, acidified water-induced alterations.

## Introduction

Since pathogenic bacteria cannot grow in acidified water, acidification of drinking water to a pH between 2.5 and 3.0 is widely used to prevent the spread of bacterial diseases in animal colonies^1^. Many research institutes including e.g., the National Institute of Health, Bethesda, MD^2–4^, Harvard Medical School, Boston, MA^5–8^, Stanford University, Stanford, CA^9–11^, Cornell University, Ithaca, NY^12–14^, University of California, Los Angeles, CA^15–17^, Boston Children’s Hospital, Boston, MA^18,19^, Texas A&M University, College Station, TX^20^, and Emory University, Atlanta, GA^21^ use acidified drinking water in their animal facilities. The Jackson Laboratory, a main provider of wild type and transgenic mouse strains for the research community, also uses acidified drinking water for their mice^22^.

Although, no adverse health effect of acidified drinking water has been reported, three recent studies showed that acidified drinking water can change the microbial flora living in the gut (gut microbiota), and this change affects autoimmunity^23–25^. Furthermore, we have recently demonstrated that HCl-acidified drinking water (average pH: 2.8) received from weaning (postnatal day 21) significantly altered the behavior and gut microbiota of *Cln3*^*−/−*^ and 129S6/SvEv wild-type mice^1^. Besides HCl, sulfuric acid (H_2_SO_4_) is also used to acidify drinking water for animal colonies^26–28^. In the current study, we examined the effects of H_2_SO_4_-acidified drinking water (pH = 2.8) received from weaning on the behavior and gut microflora of 129S6/SvEv mice, and compared the results to previously obtained data from 129S6/SvEv mice that received HCl-acidified drinking water from weaning or were kept on non-acidified drinking water. To elucidate how acidified drinking water affects the physiology of 129S6/SvEv mice, we analyzed the serum and fecal metabolomes of 6-month-old mice kept on non-acidified water or having received HCl-acidified water from weaning.

Our results show that the pH of drinking water and the mode of water acidification are major environmental factors that affect the behavior, gut microbiota and metabolome of mice. Therefore, these drinking water parameters should be reported for every mouse study.

## Results

Male 129S6/SvEv mice received drinking water acidified to pH 2.8 with H_2_SO_4_ from weaning (postnatal day 21) to examine if consumption of H_2_SO_4_-acidified water results in changes of behavior and the gut microbiota similarly to that observed previously with HCl-acidified drinking water ^1^.

### Drinking water acidified with H_2_SO_4_ alters the motor behavior of 129S6/SvEv mice differently than water acidified with HCl

**Figure 1:**
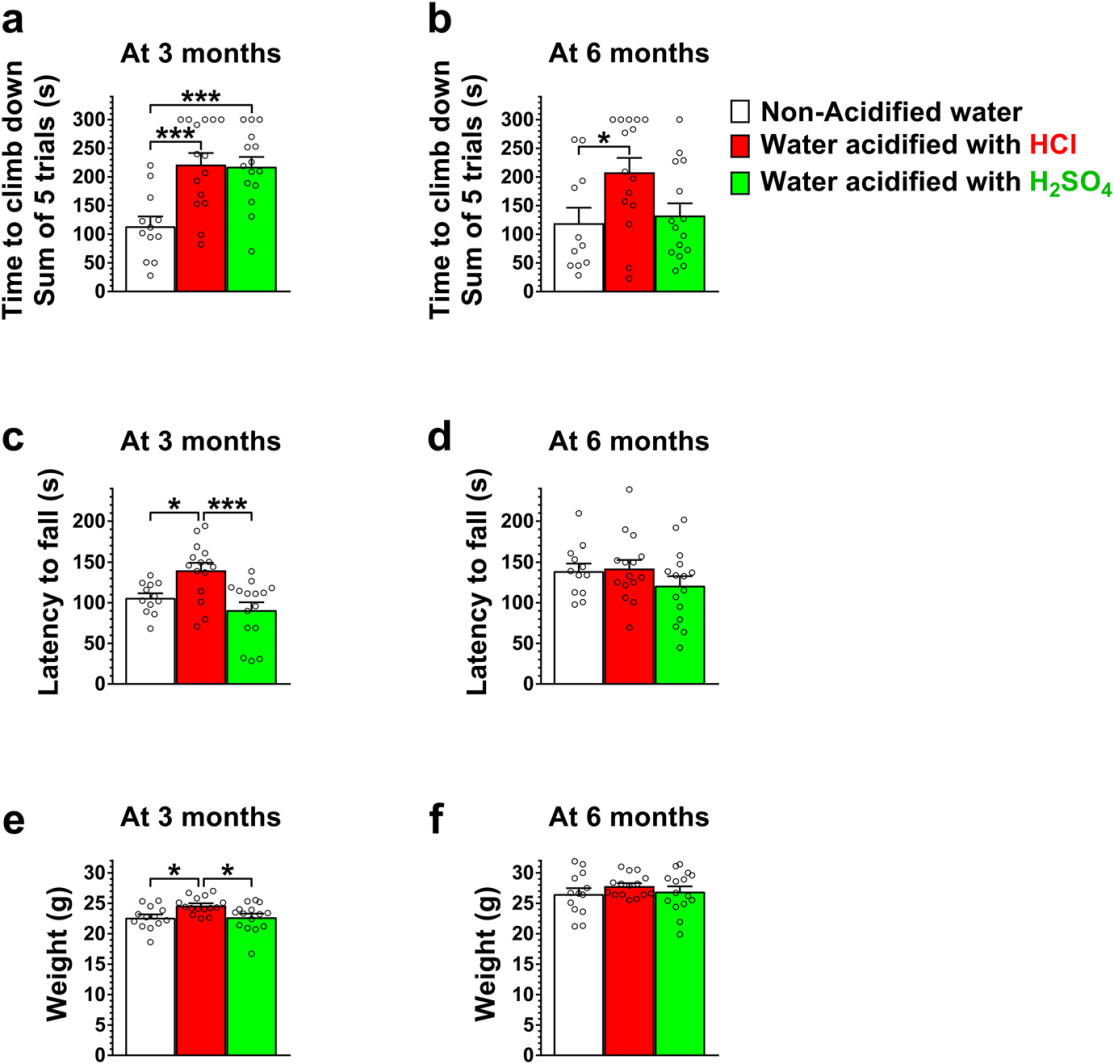
Drinking water acidified with H_2_SO_4_ alters the motor behavior of 129S6/SvEv mice differently than water acidified with HCl. 129S6/SvEv male mice were either kept on non-acidified drinking water or received drinking water acidified with H_2_SO_4_ or HCl from weaning (postnatal day 21). At the age of 3 and 6 months, mice were tested on a vertical pole to climb down and in an accelerating rotarod test (from 0 to 48 rpm in 240 s). (**a**) At 3 months of age, mice receiving H_2_SO_4_-acidified drinking water had difficulties to climb down a vertical pole, similarly to mice that received HCl-acidified drinking water. (**b**) At 6 months, the pole climbing ability of mice on H_2_SO_4_-acidified water and on non-acidified water was similar, whereas mice that received HCl-acidified water were still unable to climb down quickly. (**c**)–(**d**) H_2_SO_4_-acidified drinking water did not affect the rotarod test results at 3 and 6 months of age, whereas HCl-acidified drinking water enhanced the rotarod performance at 3 months. (**e**)–(**f**) Weight of the mice assessed in the behavioral tests at 3 (**e**) and 6 months (**f**) of age. Columns and bars represent mean ± SEM and the circles show the individual data (n = 12–15). Statistical significance was determined by 1-way ANOVA with Tukey’s post-test for multiple comparisons: *p < 0.05; ***p < 0.001.

At 3 months of age, mice receiving H_2_SO_4_-acidified drinking water had difficulties to climb down a vertical pole, similarly to mice that received HCl-acidified drinking water (Fig. 1a). At 6 months, however, the pole climbing ability of mice on H_2_SO_4_-acidified water and on non-acidified water was similar, whereas mice that received HCl-acidified water were still unable to climb down quickly (Fig. 1b). In the rotarod test, which measures balance and motor coordination, mice receiving H_2_SO_4_-acidified drinking water performed similarly to mice on non-acidified water at both 3 and 6 months of age (Fig. 1c, d). In contrast, HCl-acidified drinking water significantly enhanced the rotarod performance at 3 months (Fig. 1c). While H_2_SO_4_-acidified drinking water did not change the weight at 3 or 6 months, HCl-acidified water caused a temporary weight gain at 3 months (Fig. 1e, f).

We also tested the effect of H_2_SO_4_- or HCl-acidified drinking water on several locomotor and behavioral parameters using the Force-Plate Actimeter (BASi, West Lafayette, IN). Drinking water acidified with H_2_SO_4_ or HCl did not affect the distance traveled, left and right turn counts, spatial statistic (space utilization), bout of low mobility and focused stereotypes (head bobbing, grooming, rearing, scratching, etc.) at 3 or 6 months (Fig. S1–2). At both 3 and 6 months, the area covered, the degree of left and right turns, and the Average power (force distribution) over Band 1 (0–5 Hz) were higher in mice receiving H_2_SO_4_-acidified water but the differences did not reach statistical significance (Fig. S1–2).

### Drinking water acidified with H_2_SO_4_ alters the gut microbiota of 129S6/SvEv mice differently than water acidified with HCl

Since the gut microbiota can influence neurological functions, we examined how H_2_SO_4_-acidified drinking water affects the gut flora in comparison to HCl-acidified water. The gut microbiota composition of male 129S6/SvEv mice receiving H_2_SO_4_-acidified drinking water from weaning was determined from fecal samples by 16S rRNA gene sequencing, and analyzed together with gut microbiota data previously obtained from male 129S6/SvEv mice that were kept on non-acidified water or received HCl-acidified water from weaning^1^. Thus, the fecal DNA samples from mice receiving H_2_SO_4_-acidified drinking water were sequenced separately from the samples from mice on non-acidified and HCl-acidified water. Sequencing platforms including the Illumina MiSeq we used has sequencing errors, and separate sequencing runs may contribute to differences in the gut microbiota compositions. However, the reproducibility of Illumina MiSeq sequencing for microbiota analysis have been evaluated and high reproducibility was shown by Caporaso et al*.*^29^, Eriksson et al*.*^30^, Teng et al*.*^31^ and Antosca et al*.*^32^. We also found that mouse fecal samples collected, sequenced and analyzed 3 years apart had very similar microbiota compositions (Fig. S3), demonstrating the reproducibility of our gut microbiota analysis with DNA extraction, sequencing and analysis protocols kept identical.

**Figure 2:**
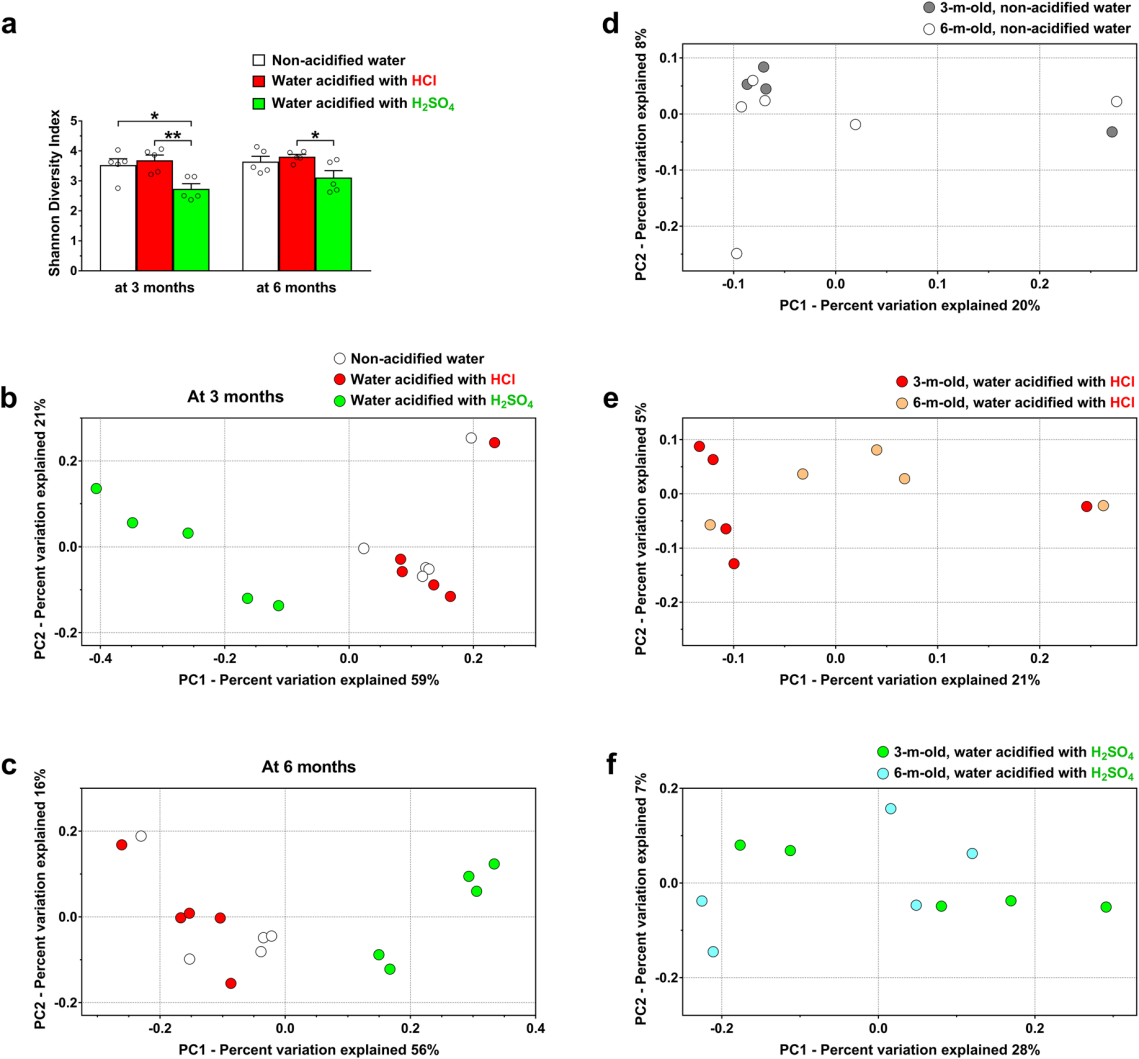
Drinking water acidified with H_2_SO_4_ alters the gut microbiota of 129S6/SvEv mice differently than water acidified with HCl. 129S6/SvEv male mice were either kept on non-acidified drinking water or received drinking water acidified with H_2_SO_4_ or HCl from weaning (postnatal day 21). Fecal pellets were collected at 3 and 6 months of age to analyze the gut microbiota by 16S rRNA gene sequencing. (**a**) Alpha diversity, the microbial diversity within a sample quantified by the Shannon diversity index, was significantly decreased by H_2_SO_4_-acidified drinking water at 3 months of age. Columns and bars represent mean ± SEM and the circles show the individual data (n = 5 mice, from 4 to 5 different cages for each group). Statistical significance was determined by 1-way ANOVA with Tukey’s post-test for multiple comparisons: *p < 0.05; **p < 0.01. (**b**)–(**c**) The bacterial community structure in mice receiving H_2_SO_4_-acidified drinking water was markedly different than the community structure in mice on non-acidified water or receiving HCl-acidified water at both 3 and 6 months of age (vs. non-acidified water: *p* = 0.024 at 3 months and *p* = 0.024 at 6 months; vs. HCl-acidified water: *p* = 0.027 at 3 months and *p* = 0.018 at 6 months). (**d**)–(**f**) Aging (3 vs. 6 months) did not result in statistically significant alterations in the overall gut microbiota composition of mice. However, some extent of age-dependent clustering was observed in mice receiving HCl-acidified water (**e**). Statistical significance in the beta diversity analyses was determined by the nonparametric statistical method PERMANOVA in R package “vegan”.

At 3 months of age, H_2_SO_4_-acidified drinking water markedly reduced alpha diversity (the microbial diversity within a sample) as compared to both non-acidified water and HCl-acidified water (Fig. 2a). At 6 months of age, alpha diversity was still significantly lower in mice receiving H_2_SO_4_-acidified water than in mice consuming HCl-acidified water (Fig. 2a). The overall composition of the bacterial community in the different groups (beta diversity) was characterized by quantifying similarities based on phylogenetic distances using UniFrac metric. Principal coordinates analysis of 16SrRNA sequence data showed strikingly different clustering of the gut microbial community in mice on H_2_SO_4_-acidified drinking water compared to mice on HCl-acidified or non-acidified water at both 3 and 6 months of age (Fig. 2b, c; vs. non-acidified water: *p* = 0.024 at 3 months and *p* = 0.024 at 6 months; vs. HCl-acidified water: *p* = 0.027 at 3 months and *p* = 0.018 at 6 months).). The gut microbiota on HCl-acidified water and non-acidified water separated to some extent at 6 months (Fig. 2c). Although aging (3 vs. 6 months) did not result in statistically significant alterations in the overall gut microbiota composition of mice, some extent of age-dependent clustering was observed in mice receiving HCl-acidified water (Fig. 2d–f).

**Figure 3:**
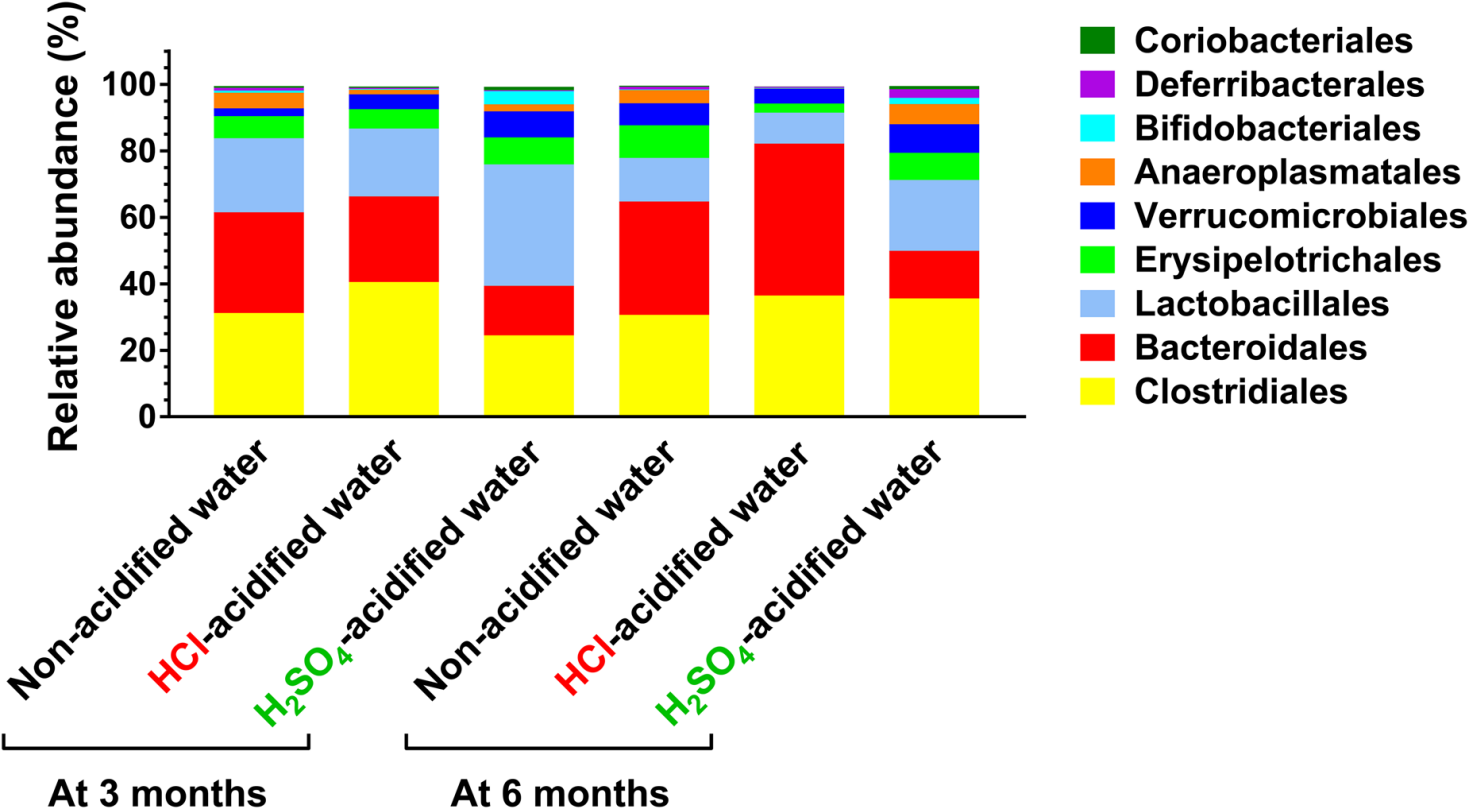
Representation of bacterial orders in the gut microbiota of 129S6/SvEv male mice kept on non-acidified drinking water or received drinking water acidified with H_2_SO_4_ or HCl from weaning (postnatal day 21). Fecal pellets were collected at three and six months of age to analyze the gut microbiota by16S rRNA gene sequencing. The stacked bar plot shows the percent composition of the gut microbiota at the order taxonomic level (averaged from 5 mice from 4 to 5 different cages for each group).

**Figure 4:**
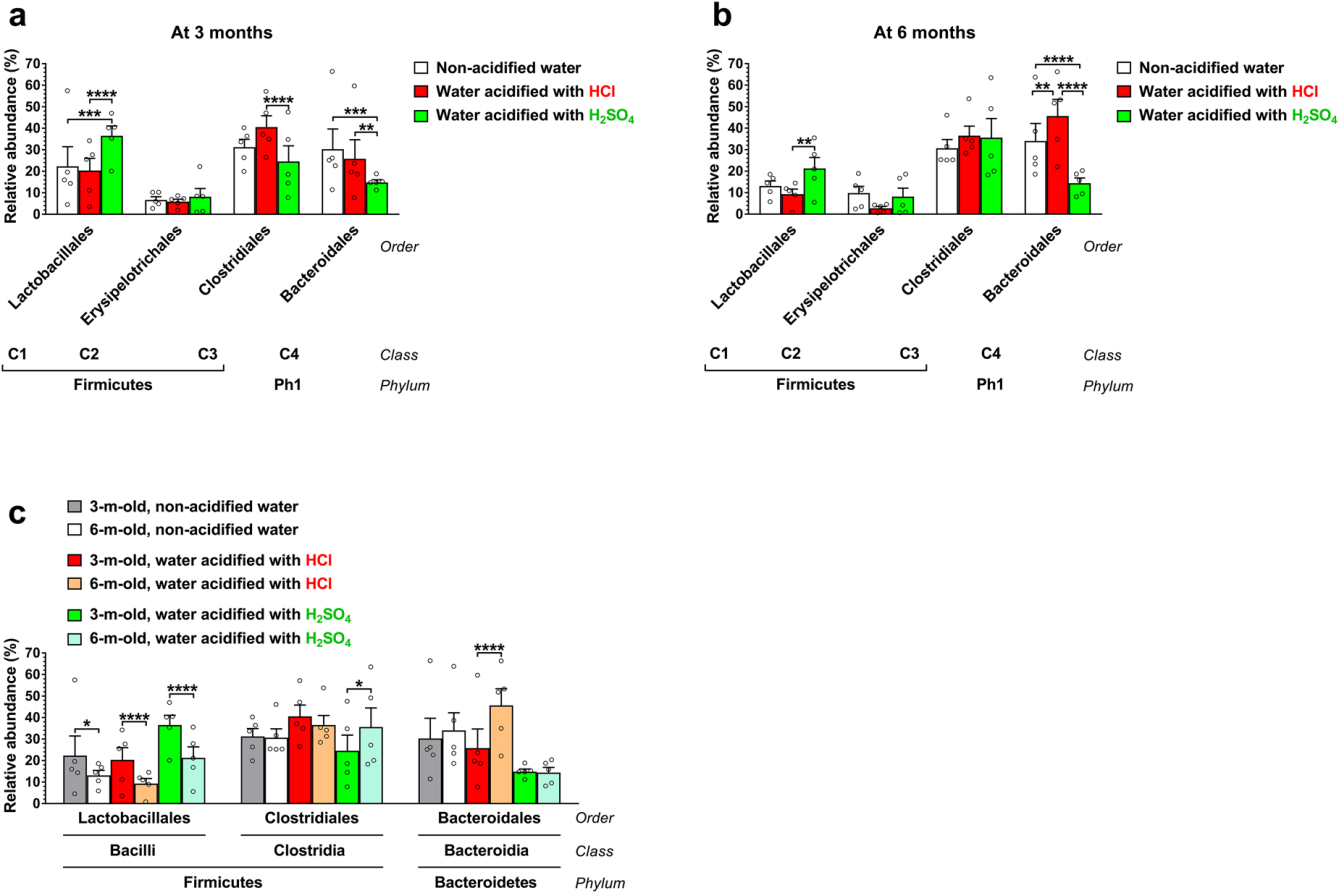
Drinking water acidified with H_2_SO_4_ alters the order composition of the gut microbiota differently than water acidified with HCl. 129S6/SvEv male mice were either kept on non-acidified drinking water or received drinking water acidified with H_2_SO_4_ or HCl from weaning (postnatal day 21). Fecal pellets were collected at 3 and 6 months of age to analyze the gut microbiota by 16S rRNA gene sequencing. (**a**)–(**b**) Differential effects of H_2_SO_4_-acidified and HCl-acidified drinking waters on the gut microbiota composition at the order taxonomic level at 3 (**a**) and 6 months (**b**) of age. (**c**) Age-dependent changes (from 3 to 6 months) in the order composition of the gut microbiota. Columns and bars represent mean ± SEM and the circles show the individual data (n = 5 mice, from 4 to 5 different cages for each group). Statistical significance was determined by 2-way ANOVA with Bonferroni’s post-test for multiple comparisons: *p < 0.05; **p < 0.01; ***p < 0.001; ****p < 0.0001. Ph1, *Bacteroidetes*; C1, *Bacilli*; C2, *Erysipelotrichia*; C3, *Clostridia*; C4, *Bacteroidia*.

We also compared the gut microbiota compositions at different taxonomic levels. The two major phyla in the human and mouse gut microbiota are *Firmicutes* and *Bacteroidetes*. While HCl-acidified water did not cause any alteration in the phylum composition, H_2_SO_4_-acidified drinking water markedly decreased the relative abundance of *Bacteroidetes* at 3 months (14.8%) as compared to non-acidified water (30.3%, *p* < 0.05) and also at 6 months (14.4%) in comparison to both non-acidified and HCl-acidified water (34.1% and 45.6%, *p* < 0.0001) (Fig. S4). At the class taxonomic level, H_2_SO_4_-acidified drinking water increased the proportion of *Bacilli* at 3 months (36.9% vs. 22.4% on non-acidified water, *p* < 0.05) and reduced the relative abundance of *Bacteroidia* at both 3 and 6 months (14.8% and 14.4% vs. 30.3% and 34.1% on non-acidified water, *p* < 0.01) (Fig. S5a–b). HCl-acidified water only caused an increase in the proportion of *Bacteroidia* at 6 months (45.6% vs. 34.1% on non-acidified water, *p* < 0.05). At the order taxonomic level, H_2_SO_4_-acidified drinking water significantly elevated the proportion of *Lactobacillales* at 3 months and reduced the relative abundance of *Bacteroidales* at both ages (Figs. 3 and 4a, b). In contrast, HCl-acidified water increased the relative abundance of *Bacteroidales* at 6 months (Figs. 3 and 4b). Significant age-dependent changes (from 3 to 6 months) were observed in the HCl-acidified water group in two phyla (*Firmicutes*, *Bacteroidetes*; Fig. S4c), two classes (*Bacilli*, *Bacteroidia*; Fig. S5c) and in two orders (*Lactobacillales*, *Bacteroidales*; Fig. 4c); in the H_2_SO_4_-acidified water group in one class (*Bacteroidia*; Fig. S5c) and in two orders (*Lactobacillales*, *Clostridiales*, Fig. 4c), and in the non-acidified water group in one order (*Lactobacillales*; Fig. 4c).

**Figure 5:**
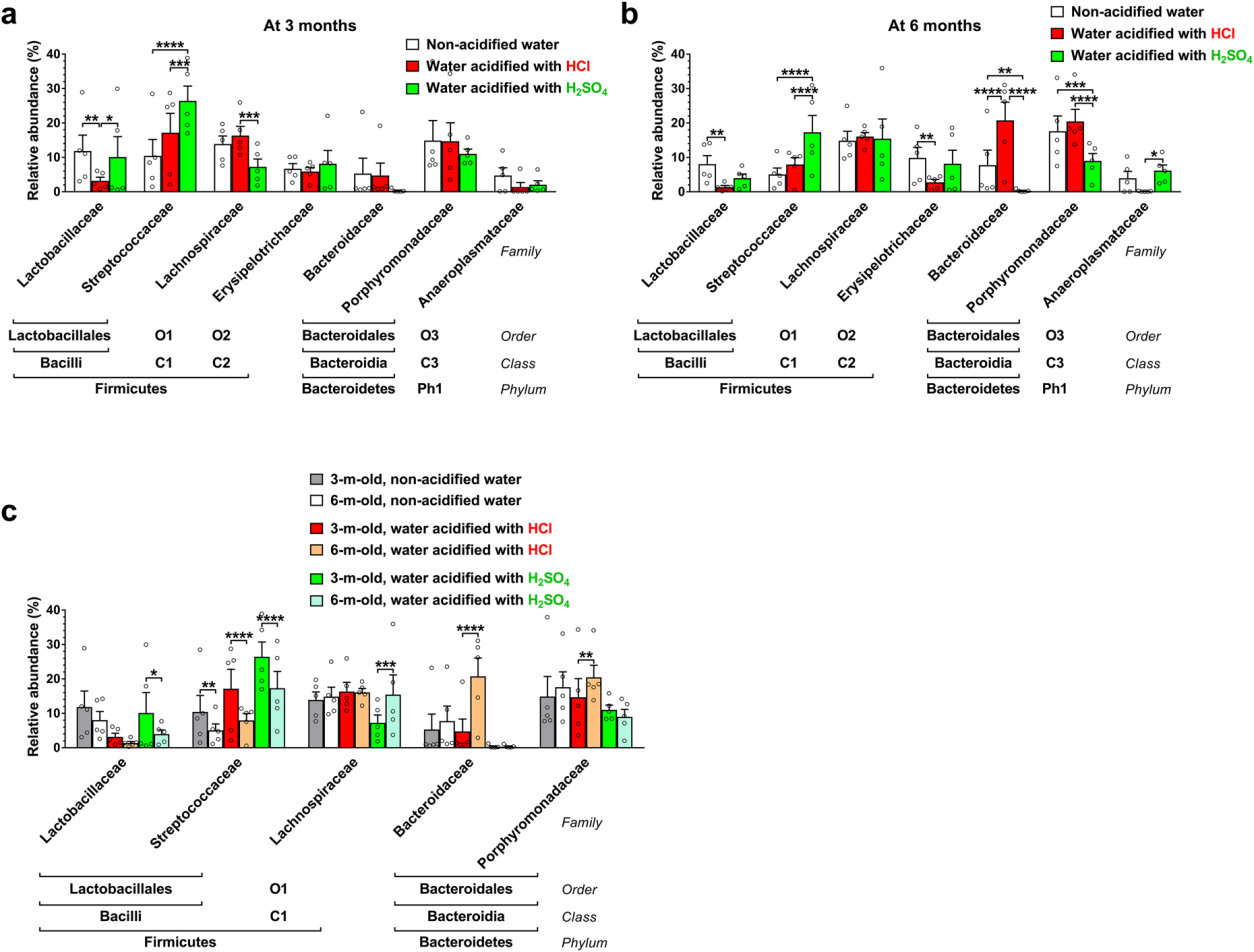
Drinking water acidified with H_2_SO_4_ changes the family composition of the gut microbiota differently than water acidified with HCl. 129S6/SvEv male mice were either kept on non-acidified drinking water or received drinking water acidified with H_2_SO_4_ or HCl from weaning (postnatal day 21). Fecal pellets were collected at 3 and 6 months of age to analyze the gut microbiota by 16S rRNA gene sequencing. (**a**)–(**b**) Differential effects of H_2_SO_4_-acidified and HCl-acidified drinking waters on the gut microbiota composition at the family taxonomic level at 3 (**a**) and 6 months (**b**) of age. (**c**) Age-dependent changes (from 3 to 6 months) in the family composition of the gut microbiota. Columns and bars represent mean ± SEM and the circles show the individual data (n = 5 mice, from 4 to 5 different cages for each group). Statistical significance was determined by 2-way ANOVA with Bonferroni’s post-test for multiple comparisons: *p < 0.05; **p < 0.01; ***p < 0.001; ****p < 0.0001. Ph1, *Tenericutes*; C1, *Clostridia*; C2, *Erysipelotrichia*; C3, *Mollicutes*; O1, *Clostridiales*; O2, *Erysipelotrichales*; O3, *Anaeroplasmatales*.

At the family taxonomic level, H_2_SO_4_-acidified drinking water significantly altered the relative abundance of *Streptococcaceae* at 3 and 6 months and of *Bacteroidaceae* and *Porphyromonadaceae* at 6 months (Fig. 5a, b). HCl-acidified drinking water caused significant changes in the proportion of *Lactobacillaceae* at both 3 and 6 months and of *Bacteroidaceae* at 6 months (Fig. 5a, b). At the genus level, H_2_SO_4_-acidified drinking water markedly altered the relative abundance of *Alistipes* and *Akkermansia* at 3 months, of *Lactococcus* and *Bacteroides* at both 3 and 6 months, and of *Lactobacillus* at 6 months (Fig. S6). HCl-acidified water significantly altered the proportion of *Lactococcus* at 3 months, of *Lactobacillus* at both 3 and 6 months, and of *Turicibacter*, *Alistipes*, *Bacteroides* and *Barnesiella* at 6 months (Fig. S6). At the family and genus taxonomic levels, age-dependent changes (from 3 to 6 months) in the gut microbiota composition occurred in all 3 groups, although, it was less frequent in mice receiving non-acidified drinking water (Fig. 5c and Fig. S7).

### Acidified drinking water causes significant alterations in the fecal and serum metabolomes of 129S6/SvEv mice

**Figure 6:**
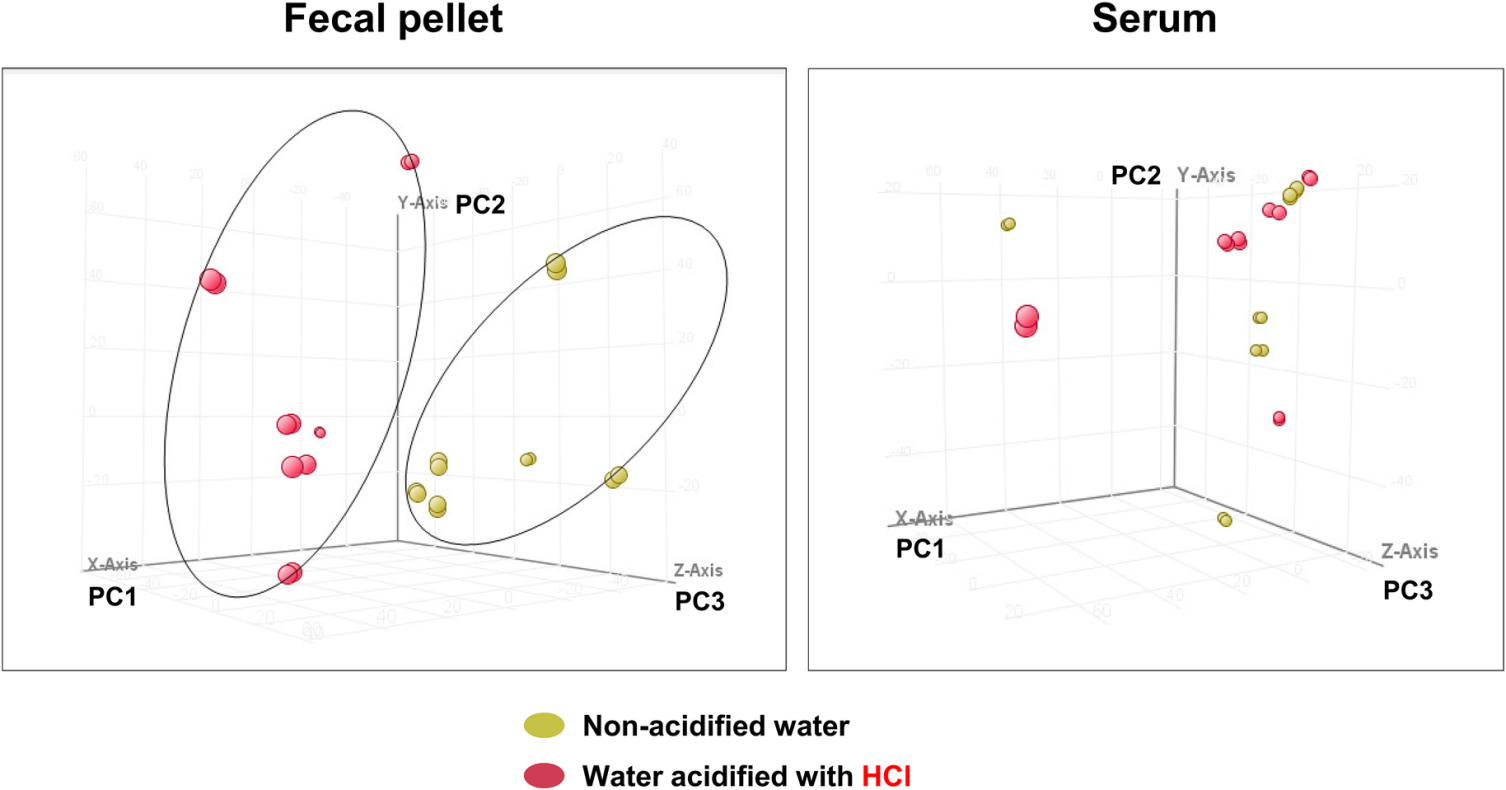
Comparative analysis of the fecal and serum metabolomes of mice kept on non-acidified water and mice that received HCl-acidified water from weaning (postnatal day 21). Fecal and serum samples were compared at 6 months of age using untargeted/global metabolomic analysis. HCl-acidified drinking water markedly changed the global fecal metabolome as shown by clear group separation using principal component analysis. HCl-acidified water had no significant effect on the global serum metabolome: principal component analysis did not show group separation. The double symbols represent two separate sample analyses from an individual mouse (n = 6 mice).

To identify pathways and mechanisms of how acidified drinking water alters the physiology of 129S6/SvEv mice, we used untargeted/global metabolomic analysis. The fecal and serum metabolomes of mice kept on non-acidified water and mice that received HCl-acidified water from weaning were compared at 6 months of age. HCl-acidified drinking water markedly changed the global fecal metabolome as shown by clear group separation using principal component analysis (Fig. 6). Table 1 shows selected fecal metabolites that had large, acidified water-induced changes in their levels (141- to 6129-fold), such as fatty acids, glycerophospholipids, a bile acid, a metabolite of the steroid hormone cortisol, an antioxidant disaccharide, the hormone and neurotransmitter adrenaline, the lysophospholipid receptor antagonist N-palmitoyl serine, a metabolite of thromboxane A2, and a metabolite of the cholinesterase inhibitor and neurotransmitter tyramine. Supplementary Table 1 lists all the identified fecal metabolites.

**Table 1:** Selected fecal metabolites with large, HCl-acidified drinking water-induced level changes (n = 6 mice).

Molecule	Molecule description	Fold change by acidified water	*p* value
7alpha-hydroxy-5beta-cholan-24-oic acid	A bile acid	140.7	0.00467
6beta-hydroxycortisol	A metabolite of the steroid hormone, cortisol	334.1	0.00244
2-Methyl-3-oxo-propanoic acid	An intermediate in the metabolism of propionic acid, a short-chain fatty acid produced and secreted by gut bacteria. 2-methyl-3-oxo-propanoic acid is a substrate for the mitochondrial enzymes, 3-hydroxyisobutyrate dehydrogenase, alanine-glyoxylate aminotransferase 2 and methylmalonate-semialdehyde dehydrogenase	492.4	0.0024
I-123 BMIPP	It is iodofiltic acid (123I), a long-chain fatty acid	2620.1	0.000002
Lactobionic acid	A disaccharide formed between beta-d-galactose and d-gluconic acid. It has a role as an antioxidant	-251.9	0.0009
l-Adrenaline	Also known as epinephrine. A hormone secreted by the adrenal glands and a neurotransmitter in the brain. It stimulates both alpha and beta adrenergic receptors	419.1	0.0023
*N*-palmitoyl serine	A selective competitive antagonist of the lysophospholipid receptors, a class of G protein-coupled plasma membrane receptors with growth factor-like effects	1351.9	0.000034
PA(18:3(6Z,9Z,12Z)/0:0)	1-(6Z, 9Z, 12Z-octadecatrienoyl)-glycero-3-phosphate, a glycerophospholipid	2399.3	0.000005
PE(18:1(9Z)/19:1(9Z))	1-(9Z-octadecenoyl)-2-(9Z-nonadecenoyl)-glycero-3-phosphoethanolamine, a glycerophospholipid	1732.6	0.000001
PI(22:6(4Z,7Z,10Z,13Z,16Z,19Z)/0:0)	1-(4Z,7Z,10Z,13Z,16Z,19Z-docosahexaenoyl)-glycero-3-phospho-(1′-myo-inositol), a glycerophospholipid	3019.8	0.000002
PS(P-16:0/15:1(9Z))	1-(1Z-hexadecenyl)-2-(9Z-pentadecenoyl)-glycero-3-phosphoserine, a glycerophospholipid	419.1	0.0017
Trans-EKODE-(E)-Ib	Trans-12,13-Epoxy-11-oxo-trans-9-octadecenoic acid, a fatty acid	1089.8	0.0001
Thromboxane B2	An inactive metabolite of thromboxane A2, which is involved in platelet activation and aggregation in case of a wound	1169.6	0.0001
Tyramine-O-sulfate	A sulfate derivative of tyramine. Tyramine is a cholinesterase inhibitor and acts as a neurotransmitter via a G protein-coupled receptor with high affinity for tyramine called TA1	6129.0	0.000024

Molecule descriptions are from PubChem (https://pubchem.ncbi.nlm.nih.gov/) and in the case of *N*-palmitoyl-serine from Liliom et al*.*^74^. *P* values were calculated using unpaired t-test with Benjamini–Hochberg correction for multiple testing.

HCl-acidified water had no significant effect on the global serum metabolome: principal component analysis did not show group separation (Fig. 6). The level of several individual serum metabolites, however, was prominently increased or reduced by HCl-acidified drinking water (3.1- to 2213-fold; Table 2). These included amino acids, fatty acids, glycerophospholipids, other lipids, and metabolites from gut bacteria. Supplementary Table 2 lists all the identified serum metabolites.

**Table 2:** HCl-acidified drinking water induced prominent changes in serum metabolite levels at 6 months of age (n = 6 mice).

Molecule	Molecule description	Fold change by acidified water	*p* value
**Amino acids and metabolites**			
Arginine		133.4	0.0238
d-Phenyllactic acid	A product of phenylalanine catabolism. The d-form of this organic acid is typically derived from bacterial sources. Levels of phenyllactic acid are normally very low in blood	296.7	0.0125
Hippuric acid	Produced by the conjugation of benzoic acid and glycine. It is a metabolite of aromatic compounds from food	− 154.4	0.0155
Homocysteine sulfinic acid	It is involved in many metabolic pathways including trans-sulfuration in cysteine synthesis, re-methylation in methionine synthesis, trans-methylation of DNA, proteins, and lipids, and biosynthesis of small hormonal and neuronal signaling molecules	− 12.8	0.0461
Indoxylsulfuric acid	A dietary protein metabolite and also a metabolite of the common amino acid tryptophan	319.1	0.0158
Urocanic acid	A deamination product of histidine. In the liver, urocanic acid is an intermediate in the conversion of histidine to glutamic acid	− 3.1	0.0054
**Fatty acids and metabolites**			
2,5-Dichloro-4-oxohex-2-enedioate	This compound belongs to the family of Medium-chain Keto Acids and Derivatives. It is a halogenated fatty acid	− 51.5	0.0399
Palmitic amide	A primary fatty acid amide derived from Palmitic acid (C16:0). Fatty acid amides compete with endocannabinoids for binding to the active site of fatty acid amide hydrolase and thus, increase the concentration of endocannabinoids by preventing their degradation	43.4	0.0382
*N*-isovalerylglycine	An acyl glycine. Acyl glycines are normally minor metabolites of fatty acids. However, the excretion of certain acyl glycines is increased in disorders associated with mitochondrial fatty acid beta-oxidation	26.5	0.0417
Methyl 2-benzamidoacetate	Also known as methylhippuric acid. It is an acyl glycine, see above	− 88.1	0.0203
Methyl-10-hydroperoxy-8E,12Z,15Z-octadecatrienoate	A hydroperoxy fatty acid	155.9	0.0127
**Glycerophospholipids**			
LysoPC(22:5(7Z,10Z,13Z,16Z,19Z))	A lysophosphatidylcholine formed by hydrolysis of phosphatidylcholine by the enzyme phospholipase A2. Lysophospholipids have a role in lipid signaling by acting on G protein-coupled lysophospholipid receptors	36.2	0.0479
LysoPE(20:4(8Z,11Z,14Z,17Z)/0:0)	A lysophosphatidylethanolamine, a breakdown product of phosphatidylethanolamine	− 24.8	0.0347
PC(14:1(9Z)/20:2(11Z,14Z))	A phosphatidylcholine with one chain of myristoleic acid at the C-1 position and one chain of eicosadienoic acid at the C-2 position	− 146.1	0.0281
PE(18:0/0:0)	1-octadecanoyl-sn-glycero-3-phosphoethanolamine, a lysophosphatidylethanolamine	− 52.8	0.0167
PE(18:3(6Z,9Z,12Z)/0:0)	1-(6Z,9Z,12Z-octadecatrienoyl)-glycero-3-phosphoethanolamine, a lysophosphatidylethanolamine	−20.9	0.0363
PE(18:4(6Z,9Z,12Z,15Z)/0:0)	1-(6Z,9Z,12Z,15Z-octadecatetraenoyl)-glycero-3-phosphoethanolamine, a lysophosphatidylethanolamine	− 19.6	0.0344
PE(19:1(9Z)/20:5(5Z,8Z,11Z,14Z,17Z))	1-(9Z-nonadecenoyl)-2-(5Z,8Z,11Z,14Z,17Z-eicosapentaenoyl)-glycero-3-phosphoethanolamine, a diacylglycerophosphoethanolamine	− 13.3	0.0414
PE(20:4(8Z,11Z,14Z,17Z)/P-16:0)	1-eicsoatetraenoyl-2-(1-enyl-palmitoyl)-sn-glycero-3-phosphoethanolamine	30.6	0.0346
PE(24:1(15Z)/22:6(4Z,7Z,10Z,13Z,16Z,19Z))	A glycerophosphoethanolamine with one chain of nervonic acid at the C-1 position and one chain of docosahexaenoic acid at the C-2 position	− 81.3	0.0242
PE(O-16:0/17:2(9Z,12Z))	1-hexadecyl-2-(9Z,12Z-heptadecadienoyl)-glycero-3-phosphoethanolamine, an acylglycerophosphoethanolamine	144.4	0.0085
PE(P-18:0/17:2(9Z,12Z))	1-(1Z-octadecenyl)-2-(9Z,12Z-heptadecadienoyl)-glycero-3-phosphoethanolamine, an acylglycerophosphoethanolamine	62.9	0.0110
PG(P-20:0/21:0)	1-(1Z-eicosenyl)-2-heneicosanoyl-glycero-3-phospho-(1′-sn-glycerol), an acylglycerophosphoglycerol	56.4	0.0119
PI(20:4(5Z,8Z,11Z,14Z)/0:0)	1-(5Z,8Z,11Z,14Z-eicosatetraenoyl)-sn-glycero-3-phospho-(1′-myo-inositol), a monoacylglycerophosphoinositol	− 20.2	0.0423
PS(O-20:0/18:0)	1-eicosyl-2-octadecanoyl-glycero-3-phosphoserine, a 1-alkyl,2-acylglycerophosphoserine	− 20.9	0.0309
**Other lipids**			
(22R)-3alpha,7alpha,22-trihydroxy-5beta-cholan-24-oic acid	A C24 bile acid	− 472.7	0.00362
3alpha-Hydroxy-5beta-chola-7,9(11)-dien-24-oic Acid	A C24 bile acid	− 160.4	0.00317
DG(18:1(9Z)/15:0/0:0)	1-oleoyl-2-pentadecanoyl-sn-glycerol, a diacylglycerol	84.9	0.0388
Phytosphingosine	A sphingoid, structurally similar to sphingosine, which is a highly bioactive compound. The physiological roles of phytosphingosine are largely unknown	146.8	0.0129
4α-formyl-4β-methyl-5α-cholesta-8-en-3β-ol	A sterol lipid molecule	58.6	0.0322
*N*-stearoyl glutamic acid	Belongs to the *N*-acyl amino acid family of bioactive lipids	− 29.5	0.0192
**Miscellaneous**			
Bichanin A 7-O-glucoside-6′'-malonate	It is a flavonoid and gut bacteria have a crucial role in the metabolism of dietary flavonoids	72.7	0.0190
Demethyltexasin	4′,6,7-Trihydroxyisoflavone, metabolized by gut bacteria from daidzein, an isoflavone compound in food plants. It has a role as a peroxisome proliferator-activated receptor (PPAR) alpha agonist, a PPARgamma agonist, an anti-inflammatory agent, and an antimutagen	82.2	0.0350
d-Gal alpha 1->6d-Gal alpha 1->6d-Glucose	A trisaccharide	98.0	0.0184
*N*-cis-tetradec-9Z-enoyl-l-Homoserine lactone	A long-chain *N*-acylated homoserine lactone. Acyl homoserine lactones mediate bacterial cell–cell communication, also known as quorum sensing, in order to monitor and respond to microbial density	91.2	0.0283
l-Homoserine lactone	l-homoserine lactone is a product of acyl-homoserine lactone metabolism. Acyl homoserine lactones mediate bacterial cell–cell communication, also known as quorum sensing, in order to monitor and respond to microbial density	− 27.2	0.0383
*N*,*N*-Diacetylchitobiosyldiphosphodolichol	A polyprenyl phospho oligosaccharide metabolite	42.9	0.0293
*N*-Glycolylneuraminic acid	A sialic acid molecule (an amino sugar) used in the Golgi for glycosylation	− 2213	0.000019
Trimethylaminoacetone	Gut bacteria can metabolize carnitine to trimethylamine and possibly to trimethylaminoacetone	− 41.2	0.0203
Tyr-Trp-Cys	A tripeptide with radical scavenging activity	33.5	0.0300
1-Methylhypoxanthine	A methylated hypoxanthine. Hypoxanthine is a naturally occurring purine derivative and a reaction intermediate in the metabolism of adenosine and in the formation of nucleic acids by the salvage pathway	− 75.1	0.0193

Molecule descriptions are from PubChem (https://pubchem.ncbi.nlm.nih.gov/) and in the case of *N*-cis-tetradec-9Z-enoyl-l-Homoserine lactone and l-Homoserine lactone from Leadbetter and Greenberg^75^. *P* values were calculated using unpaired t-test with Benjamini–Hochberg correction for multiple testing.

## Discussion

Our study demonstrates that the effects of acidified drinking water on the behavior and gut microbiota of control, wild-type (129S6/SvEv) mice depends on the acid used for acidification. H_2_SO_4_-acidified drinking water had a much stronger effect on the gut microbiota composition than HCl-acidified water, most likely due to the important role of sulfur in bacterial metabolism. In gut bacteria, the sulfate from H_2_SO_4_ can be reduced by assimilatory sulfate reduction to elemental sulfur resulting in increased protein synthesis and growth^33,34^. Moreover, intestinal sulfate-reducing bacteria use sulfate as an electron acceptor in energy production with an end product of hydrogen sulfide that is released and can be toxic to various bacteria, inducing a shift in the gut microbiota composition^33,35^.

Since we did not analyze the gut microbiota of mice before they started receiving HCl- or H_2_SO_4_-acidified drinking water at weaning (postnatal day 21), the contribution of potential differences in the basal gut microbiota composition to the acidified drinking water-induced effects cannot be ruled out. Large differences in the basal gut microbiota composition, however, are unlikely. First, the mouse gut microbiota, which is primarily determined by the genetic background^36,37^, is stable for a year post-weaning if environmental factors such as diet, bedding material and cage type are strictly controlled^38^. This was the case in our study. 129S6/SvEv mice were maintained in our mouse colony, in the same room, in individually vented microisolator cages with corn cob bedding, under identical conditions, receiving the same diet, and drinking water (non-acidified, HCl- or H_2_SO_4_-acidified) was the only variable. Furthermore, the extent of the difference in the global gut microbiota composition (beta diversity) between 129S6/SvEv mice receiving H_2_SO_4_-acidified or non-acidified drinking water (Fig. 2) is similar to that we recently found between C57BL/6J and 129S6/SvEv mice, two very distinct mouse strains^39^. This magnitude of the H_2_SO_4_-acidified water-induced changes is beyond any possible interindividual variations in the gut microbiota among 129S6/SvEv mice. Moreover, in all three experimental groups in our study, fecal samples from the same mice were analyzed at both 3 and 6 months of age. In contrast to mice on non-acidified water, the gut microbiota composition of mice receiving HCl- or H_2_SO_4_-acidified drinking water prominently changed from 3 to 6 months at every taxonomic rank, indicating a clear effect of the prolonged exposure to acidified water in the same mice.

Although, H_2_SO_4_-acidified drinking water altered the gut microbiota of 129S6/SvEv mice more severely than HCl-acidified water, the latter caused more pronounced changes in neurological functions. Searching for a potential explanation for the H_2_SO_4_- and HCl-acidified drinking water-induced behavioral changes, we examined if any alteration in the gut microbiota at the individual taxonomic ranks correlates with the pole climbing and rotarod test results. No consistent correlations were found, indicating that individual changes in the gut microbiota composition alone are not responsible for the acidified water-induced anomalies in motor function.

In mice consuming HCl-acidified drinking water, the increased chloride ion concentration in the gut probably has direct effects on intestinal physiology by affecting various epithelial chloride channels^40–42^. The altered state of gastrointestinal epithelial cells may influence the signals sent to the brain through the chemosensitive vagus nerve afferents^43^. The vagal afferent pathway is able to activate/regulate the hypothalamic–pituitary–adrenal axis^44^, and thus affects behavior. Through epithelial chloride channels, chloride ions from the HCl-acidified drinking water may also modulate the function of intestinal enteroendocrine cells, which release more than 30 gastrointestinal neurohormones and several of them act centrally to modulate the activity of brainstem vagal neurons^43^.

To shed some light on how the most widely used HCl-acidified drinking water affects the physiology of 129S6/SvEv mice, we analyzed the serum and fecal metabolomes and found remarkable, acidified water-induced alterations. In the fecal metabolome, the level of adrenaline was increased 419-fold. Several gut bacteria can produce noradrenaline^45^, which is converted to adrenaline in the adrenal medulla, and adrenaline, both as a hormone and neurotransmitter, affects animal behavior^46,47^. The level of 6β-hydroxycortisol, a metabolite of the stress hormone cortisol, was increased 334-fold. Gut bacteria metabolize cortisol and those metabolites, when reabsorbed in the intestine back into the circulation, produce biological effects^48^. It has been shown that gut bacteria produce the neurotransmitter tyramine^49^. The highly increased level of its metabolite, tyramine-O-sulfate, in fecal samples of mice consuming HCl-acidified drinking water indicates altered tyramine synthesis and metabolism, which can affect gastrointestinal physiology and neurological function^50^.

HCl-acidified drinking water also caused pronounced alterations in the serum metabolome. Serum arginine level was elevated 133-fold, which indicates increased production of nitric oxide and polyamines^51–54^ that are important modulators of neuronal activity^55,56^. HCl-acidified drinking water caused a 43-fold increase in the serum level of palmitic amide, a primary fatty acid amide derived from palmitic acid (C16:0). Fatty acid amides are neuroactive lipids and it has been suggested that they compete with endocannabinoids for binding to fatty acid amide hydrolase and therefore, increase the concentration of endocannabinoids by preventing their degradation^57^. Endocannabinoid signaling has an important role in motor control^58^.

Among glycerophospholipids, a lysophosphatidylcholine has increased serum level whereas several lysophosphatidylethanolamines had markedly reduced serum levels, and these changes may also contribute to the impaired pole-descending ability of mice receiving HCl-acidified drinking water. Lysophospholipids have diverse biological effects acting on G protein-coupled lysophospholipid receptors, and in the central nervous system they regulate synaptic transmission and have various effects on astrocytes, oligodendrocytes and microglia^59^.

In summary, our result show that the pH of drinking water and the mode of water acidification are major environmental factors that strongly affect the gut microbiota, fecal and serum metabolome and neurological function of control wild-type mice commonly used in transgenic studies. To reduce the inter-laboratory variations in mouse studies, the type of drinking water should always be described in publications.

## Methods

### Animals

129S6/SvEv wild-type mice were maintained in our mouse colony as we previously described^1^. All mice were housed in the same room with a 14-h light, 10-h dark cycle. Mice were housed in individually vented microisolator cages (4–5 mice/cage) with ad libitum access to food and water. Mice were fed with the Teklad Global 2918 diet (Harlan Laboratories, Indianapolis, IN, USA), and their drinking water was either non-acidified tap water (pH 8.4), tap water acidified with HCl to pH 2.5–2.9 (average was 2.8) using a Technilab BMI BV water acidification system (Tecniplast USA, West Chester, PA, USA) or tap water acidified with H_2_SO_4_ to pH = 2.8. All animal procedures were carried out according to the guidelines of the Animal Welfare Act and NIH policies, and were approved by the Sanford Research Animal Care and Use Committee.

Our study is in compliance with the ARRIVE guidelines (Animal Research: Reporting of In Vivo Experiments)^60^.

### Behavioral testing

Behavioral testing was carried out according to our published methods^1^. Mice were transported to the behavioral testing room where the lights had been dimmed. Mice were labeled on their tails with a marker for easy identification, weighed, and were allowed to accommodate to the room at least for 20 min before starting the behavioral tests. All mice were tested first in the pole climbing test and then in the Force-Plate Actimeter. One day later the same mice were also tested in the rotarod test. The pole climbing test and rotarod test were carried out under dim light to keep the anxiety level of mice minimal. The same mice were tested at 3 and 6 months of age. To minimize the cage effect on behavior, mice from 5 to 6 different cages (12–18 mice) for each group were used for behavioral testing.

#### Pole climbing test

This test assesses the balance, spatial orientation, and motor coordination of mice, though anxiety and motivation to move may also affect the test results. The test was carried out as we previously described^61^. The vertical pole was an all-thread metal rod (diameter: 1.27 cm; height: 66 cm), screwed to a 3.81-cm-thick plastic block (24.5 cm × 25.4 cm). The plastic block was covered with a 3.81-cm-thick green hunting seat cushion (nitrile rubber/PVC foam) to prevent the mice from injury when they fell from the pole. The height of the pole measured from the surface of the hunting seat cushion was 59 cm. The mouse was placed, head downward, on top of the pole, and the time until the mouse climbed down to the base of the pole was measured in 5 consecutive trials. Each climbing down trial was terminated after 60 s to avoid exhaustion. If the mouse fell from the pole a trial result of 60 s was given. The time to climb down (sum of the 5 trials in seconds) was calculated for each mouse.

#### Force-plate actimeter

In the Force Plate Actimeter (BASi, West Lafayette, IN, USA), multiple behaviors can be studied in the same freely moving animal. General locomotor activity (distance traveled, area covered, spatial statistic, number of left and right turns), focused stereotypes (head bobbing, grooming, rearing, scratching, etc.), low mobility bouts (each defined as remaining continuously in a virtual circle of 15-mm radius for 5 s), average power (force distribution) over different frequency ranges, and tremor can be quantified^62^. Mice were tested in the Force-Plate Actimeter as we previously described^1^ with some modification. Briefly, mice were placed on the 44 × 44 cm platform of the Force-Plate Actimeter for a 10.24-min recording session, and the above-mentioned behavioral parameters were recorded. The force-plate actimeter recorded data in thirty 20.48-s frames, averaging 1024 data points in each frame. All recorded information was processed and analyzed using FPAAnalysis software version 1.10.01 (BASi, West Lafayette, IN, USA; https://www.basinc.com/assets/library/manuals/FPA.pdf).

#### Rotarod test

The rotarod uses a motor-driven, rotating rod to measure the fore- and hind limb motor coordination and balance of mice. Motor learning capability and endurance level may also affect the rotarod performance. The rotarod test using two Rotamex-5 accelerating rotarod instruments (Columbus Instruments, Columbus, OH, USA, diameter of the rotating rod: 3 cm) was performed as described previously^61^. The start speed of the rotarod was 0 rpm and the acceleration was set to 0.2 rpm/s. The cut-off time was set at 240 s. Mice were trained on the rotarod in three consecutive runs. Following training, mice rested for 1.5 h and then were tested on the rotarod in three test trials each consisting of three consecutive runs, with 15 min of rest between the trials. The average latency to fall from the rotating rod in the test trials (average of the 9 runs in the 3 trials) was calculated for each mouse.

### Analysis of the gut microbiota

#### Fecal pellet collection

Fecal pellets were collected from 3- and 6-month-old male mice (5 mice from 4 to 5 different cages for each type of drinking water) as we previously described^1^. Fecal pellets were collected with a pair of disinfected forceps in sterile 1.5-ml tubes at the end of the pole climbing test and the Force-Plate Actimeter test (each mouse was tested individually). Immediately after an individual sample collection, the tube was placed on dry ice. After each mouse, the pole climbing apparatus and the platform of the Force-Plate Actimeter were disinfected to prevent the cross-contamination of fecal samples. Collected fecal samples were stored at − 80 °C, and shipped on dry ice to MR DNA (www.mrdnalab.com, Shallowater, TX, USA) for microbiota analysis.

#### 16S rRNA gene sequencing (Illumina bTEFAP 2 × 300 bp 20 k Diversity assay)

DNA extraction from the mouse fecal pellets, 16S rRNA gene sequencing (Illumina bTEFAP 2 × 300 bp 20 k Diversity assay) and taxonomic identification were performed by MR DNA (www.mrdnalab.com, Shallowater, TX, USA) as previously described^1^. To minimize the cage effect on the gut microbiota composition, fecal pellets collected from 4 to 5 different cages (5 mice) were analyzed for each group. DNA was isolated from the mouse fecal pellets using Qiagen QIAamp DNA Stool Mini Kit (Qiagen, Valencia, CA, USA). 16S rDNA bacterial tag-encoded FLX amplicon pyrosequencing (bTEFAP), a high-throughput universal tool for bacterial diversity determination was originally described by Dowd et al.^63^ and has been utilized in a large number of studies analyzing the gut microbiota in different species (cattle, mice, pigs and humans) and environmental samples (see e.g.^64–66^.). A redesign modern version of bTEFAP adapted to the Illumina MiSeq platform was used to determine the microbiota composition in the mouse fecal samples.

The 16S rRNA gene V4 variable region PCR primers 515F (GTGYCAGCMGCCGCGGTAA) and 806R (GGACTACNVGGGTWTCTAAT) with barcode on the forward primer were used in a 30 cycle PCR (5 cycle used on PCR products) using the HotStarTaq Plus Master Mix Kit (Qiagen, Valencia, CA, USA) under the following conditions: 94 °C for 3 min, then 28 cycles of 94 °C for 30 s, 53 °C for 40 s and 72 °C for 1 min, followed by a final elongation step at 72 °C for 5 min. After amplification, PCR products were examined in a 2% agarose gel to determine the success of amplification and the relative intensity of bands. Multiple samples were pooled together (e.g., 100 samples) in equal proportions based on their molecular weight and DNA concentrations. Pooled samples were purified using calibrated Ampure XP beads. Then the pooled and purified PCR products were used to prepare DNA library according to the Illumina TruSeq DNA library preparation protocol. Sequencing was performed in an Illumina MiSeq system following the manufacturer’s guidelines. Sequence data were processed using MR DNA standardized analysis pipeline. Briefly, sequences were joined, depleted of barcodes then sequences < 150 bp and sequences with ambiguous base calls were removed. Sequences were denoised, operational taxonomic units (OTUs) were generated, and chimeras were removed using the UCHIME program^67^. OTUs were defined by clustering at 3% divergence (97% similarity). Final OTUs were taxonomically classified using BLASTn against MR DNA’s customized, proprietary database derived from the 2016 versions of RDPII (http://rdp.cme.msu.edu) and NCBI (www.ncbi.nlm.nih.gov).

#### Data analysis

The global microbiota analyses were performed using packages under R software (version 3.4.3; https://cran.r-project.org/) to identify significant differences between treatment groups based on the bacterial relative abundance profiles. Principal coordinates analysis was conducted using R package “phyloseq” version 1.30.0^68^. The significance of group separation was tested by the nonparametric statistical method PERMANOVA in R package “vegan” (version 2.5-6; https://cran.r-project.org/web/packages/vegan/index.html). Post-hoc pairwise comparisons among levels of grouping factors were performed using function “pairwise.adonis” in “vegan” package with p values adjusted for multiple comparisons.

Alpha diversity, the microbial diversity within the samples, was assessed by the Shannon diversity index, which was calculated using the function “estimate_richness” in the “phyloseq” R package. One-way ANOVA with Tukey’s post-test was used to compare Shannon indices.

The gut microbiota taxonomic data were analyzed by GraphPad Prism 7.04 (GraphPad Software, San Diego, CA, USA; www.graphpad.com).

### Untargeted/global metabolomic analysis

#### Sample collection

Fecal and serum samples were collected from the same mice at 6 months of age (6 mice from 4 to 5 different cages for each type of drinking water). Fecal pellets were collected and stored as described above in the “Analysis of the gut microbiota” section. To obtain serum samples, mice were euthanized by CO_2_ exposure and blood was collected from the right ventricle of the heart with a 22 gauge needle attached to a 1-ml syringe. The blood was immediately transferred in a sterile 1.5-ml tube, let it coagulated for 1 h at room temperature and then centrifuged at 1000*g* for 15 min at 4 °C. The serum was transferred in a sterile 1.5-ml tube and stored at − 80 °C. The collected fecal and serum samples were shipped on dry ice to the Mayo Clinic Metabolomics Core (Rochester, MN, USA) for untargeted metabolomic analysis.

#### Metabolomic analysis

^13^C_6_-phenylalanine (3 µl at 250 ng/µl) was added as internal standard to fecal homogenates (50 µl) and serum samples (25 µl) prior to deproteinization. Fecal homogenates and serum samples were deproteinized with six-times volume of cold acetonitrile:methanol (1:1 ratio), kept on ice with intermittent vortexing for 30 min at 4 °C, then centrifuged at 18,000*g*. The supernatants were divided into 4 aliquots and dried down using a stream of nitrogen gas for analysis on a Quadrupole Time-of-Flight Mass Spectrometer (Agilent Technologies 6550 Q-TOF) coupled with an Ultra High Pressure Liquid Chromatograph (1290 Infinity UHPLC Agilent Technologies). Profiling data was acquired under both positive and negative electrospray ionization conditions over a mass range m/z of 100–1700 at a resolution of 10,000 (separate runs) in scan mode. Metabolite separation was achieved using two columns of differing polarity, a hydrophilic interaction column (HILIC, ethylene-bridged hybrid 2.1 × 150 mm, 1.7 mm; Waters) and a reversed-phase C18 column (high-strength silica 2.1 × 150 mm, 1.8 mm; Waters) with gradient described previously^69–71^. Run time was 18 min for HILIC and 27 min for C18 column using a flow rate of 400 µl/min. A total of four runs per sample were performed to give maximum coverage of metabolites. Samples were injected in duplicate, wherever necessary, and a pooled quality control (pooled QC) sample, made up of all of the samples from each batch was injected several times during a run. A separate plasma quality control (QC) sample was analyzed with pooled QC to account for analytical and instrumental variability. Dried samples were stored at − 20 °C till analysis. Samples were reconstituted in running buffer and analyzed within 48 h of reconstitution. Auto-MS/MS data was also acquired with pooled QC sample to aid in unknown compound identification using fragmentation pattern.

#### Data analysis

Data alignment, filtering, univariate, multivariate statistical and differential analysis was performed using Mass Profiler Professional version 14.8 (Agilent Inc, Santa Clara, CA, USA; https://www.agilent.com/en/product/software-informatics/mass-spectrometry-software/data-analysis/mass-profiler-professional-software). Metabolites detected in at least ≥ 80% of one of two groups were selected for differential expression analyses. Metabolite peak intensities and differential regulation of metabolites between groups were determined as described previously^69,70^. Each sample was normalized to the internal standard and log 2 transformed. Unpaired t-test with multiple testing correction *p* < 0.05 was used to find the differentially expressed metabolites between two groups. Default settings were used with the exception of signal-to-noise ratio threshold^70^, mass limit (0.0025 units), and time limit (9 s). Putative identification of each metabolite was done based on accurate mass (m/z) against METLIN database using a detection window of ≤ 7 ppm. The putatively identified metabolites were annotated as Chemical Abstracts Service (CAS), Kyoto Encyclopedia of Genes and Genomes (KEGG)^72^, Human Metabolome Project (HMP) database, and LIPID MAPS identifiers^73^.

### Statistical analysis

Statistical analysis was performed using GraphPad Prism 7.04 (GraphPad Software, San Diego, CA, USA; www.graphpad.com). Most of the data sets from the pole climbing test, rotarod test, and weight measurement passed the normality test (alpha level 0.05), and therefore, 1-way ANOVA with Tukey’s post-test was used for comparison. The gut microbiota compositions at all taxonomic levels were compared by 2-way ANOVA with Bonferroni’s post-test for multiple comparisons. In the untargeted/global metabolomic analysis, statistical significance was calculated using unpaired t-test with Benjamini–Hochberg correction for multiple testing. Alpha level was 0.05 in all statistical tests.

## Supplementary Information

Supplementary Tables.

Supplementary Figures.

## Data Availability

The complete lists of identified fecal and serum metabolites are in the Supplementary Tables. The 16S rRNA gene sequencing datasets used and analyzed in the current study are available from the corresponding author on reasonable request.

## Abbreviations

OTUs: Operational taxonomic units
WT: Wild-type
