# Supplementary material for: The acidified drinking water-induced changes in the behavior and gut microbiota of wild-type mice depend on the acidification mode
Source: Sci Rep. 2021 Feb 3;11:2877. doi: 10.1038/s41598-021-82570-0 (PMC7858586; doi:10.1038/s41598-021-82570-0)
Supplement: Supplementary file 2 — Supplementary Figures. [file 41598_2021_82570_MOESM2_ESM.docx]

**The acidified drinking water-induced changes in the behavior and gut microbiota of wild-type mice depend on the acidification mode**

Brandon Whipple^1^, Jennifer Agar^1^, Jing Zhao^2,4,a^, David A. Pearce^3,5^ and Attila D. Kovács^3,5,^*

^1^Animal Resource Center, ^2^Population Health Group and ^3^Pediatrics and Rare Diseases Group at Sanford Research, Sioux Falls, South Dakota, 57104, USA; ^4^Department of Internal Medicine and ^5^Department of Pediatrics, Sanford School of Medicine, University of South Dakota, Sioux Falls, South Dakota, 57105, USA

***Corresponding author:**

Attila D. Kovács, PhD

Pediatric and Rare Diseases Group, Sanford Research

2301 E. 60^th^ Street N.,

Sioux Falls, South Dakota, 57014

Tel: +1 605-312-6404

E-mail: [Attila.Kovacs@sanfordhealth.org](mailto:Attila.Kovacs@sanfordhealth.org)

**^a^**Current address: Center for Biostatistics, Ohio State University, Columbus, Ohio, 43210, USA

**Supplementary Figures**

**Supplementary Fig. 1. Drinking water acidified with H_2_SO_4_ or HCl did not affect locomotor and behavioral parameters measured in a force-plate actimeter at 3 months of age.** 129S6/SvEv male mice were either kept on non-acidified drinking water or received drinking water acidified with H_2_SO_4_ or HCl from weaning (postnatal day 21). **At the age of 3 months**, mice were tested in a force-plate actimeter, which measures several behavioral parameters in freely moving animals. The force-plate actimeter recorded data for 10.24 minutes, in thirty 20.48-second frames, averaging 1,024 data points in each frame. **a)** Right turn count. **b)** Right turn total degree. **c)** Left turn count. **d)** Left turn total degree. **e)** Total distance traveled. **f)** Area covered. **g)** Spatial statistic (space utilization). **h)** Bout of low mobility. **i)** Focused stereotypes (head bobbing, grooming, rearing, scratching, etc.). **j)** Average power (force distribution) over band 1 (0-5 Hz). Columns and bars represent mean ± SEM and the circles show the individual data (n=12-15). No statistically significant differences were identified by 1-way ANOVA with Tukey’s post-test for multiple comparisons.

**Supplementary Fig. 2. Drinking water acidified with H_2_SO_4_ or HCl did not affect locomotor and behavioral parameters measured in a force-plate actimeter at 6 months of age.** 129S6/SvEv male mice were either kept on non-acidified drinking water or received drinking water acidified with H_2_SO_4_ or HCl from weaning (postnatal day 21). **At the age of 6 months**, mice were tested in a force-plate actimeter, which measures several behavioral parameters in freely moving animals. The force-plate actimeter recorded data for 10.24 minutes, in thirty 20.48-second frames, averaging 1,024 data points in each frame. **a)** Right turn count. **b)** Right turn total degree. **c)** Left turn count. **d)** Left turn total degree. **e)** Total distance traveled. **f)** Area covered. **g)** Spatial statistic (space utilization). **h)** Bout of low mobility. **i)** Focused stereotypes (head bobbing, grooming, rearing, scratching, etc.). **j)** Average power (force distribution) over band 1 (0-5 Hz). Columns and bars represent mean ± SEM and the circles show the individual data (n=12-15). No statistically significant differences were identified by 1-way ANOVA with Tukey’s post-test for multiple comparisons.

**Supplementary Fig. 3. Fecal samples collected, sequenced and analyzed 3 years apart have very similar microbiota compositions,** **demonstrating the reproducibility of gut microbiota analysis.** Fecal samples were collected from 3-month-old mixed 129S6/SvEv x C57BL/6J wild-type (WT) and *Cln1^R151X^* (on the mixed 129S6/SvEv x C57BL/6J background) male mice first in 2016 then, for another study in 2019. The *Cln1^R151X^* nonsense mutant mouse is a model of the childhood neurodegenerative disorder, infantile CLN1 disease. The microbiota composition of the samples was determined by16S rRNA gene sequencing in 2016 and 2019. DNA extraction, sequencing and analysis of the microbiota composition were carried out at MR DNA (www.mrdnalab.com, Shallowater, TX, USA). **a)** Phylum composition of the gut microbiota in 3-month-old WT at *Cln1^R151X^* male mice in 2016 and 2019. **b)** Class composition of the gut microbiota in 3-month-old WT at *Cln1^R151X^* male mice in 2016 and 2019. Columns and bars represent mean ± SEM and the circles show the individual data (n=5 mice; *Cln1^R151X^* in 2016: n=4 mice; from 2-4 different cages for each group).

**Supplementary Fig. 4. Only drinking water acidified with H_2_SO_4_ changed the phylum composition of the gut microbiota in 129S6/SvEv mice.** 129S6/SvEv male mice were either kept on non-acidified drinking water or received drinking water acidified with H_2_SO_4_ or HCl from weaning (postnatal day 21). Fecal pellets were collected at 3 and 6 months of age to analyze the gut microbiota by16S rRNA gene sequencing. **a-b)** H_2_SO_4_-acidified drinking water caused significant changes in the phylum composition of the gut microbiota at both 3 (a) and 6 months (b) of age. **c)** Only HCl-acidified drinking water caused age-dependent changes (from 3 to 6 months) in the phylum composition of the gut microbiota. Columns and bars represent mean ± SEM and the circles show the individual data (n=5 mice, from 4-5 different cages for each group). Statistical significance was determined by 2-way ANOVA with Bonferroni’s post-test for multiple comparisons: ^*^p<0.05, ^**^p<0.01, ^****^p<0.0001.

**Supplementary Fig. 5. Class level analysis of the gut microbiota of 129S6/SvEv male mice kept on non-acidified drinking water or received drinking water acidified with H_2_SO_4_ or HCl from weaning (postnatal day 21).** Fecal pellets were collected at 3 and 6 months of age to analyze the gut microbiota by 16S rRNA gene sequencing. **a-b)** Differential effects of H_2_SO_4_-acidified and HCl-acidified drinking waters on the gut microbiota composition at the class taxonomic level at 3 (a) and 6 months (b) of age. **c)** Age-dependent changes (from 3 to 6 months) in the class composition of the gut microbiota. Columns and bars represent mean ± SEM and the circles show the individual data (n=5 mice, from 4-5 different cages for each group). Statistical significance was determined by 2-way ANOVA with Bonferroni’s post-test for multiple comparisons: ^*^p<0.05, ^**^p<0.01, ^****^p<0.0001.

**Supplementary Fig. 6. Genus level analysis of the gut microbiota of 129S6/SvEv male mice kept on non-acidified drinking water or received drinking water acidified with H_2_SO_4_ or HCl from weaning (postnatal day 21).** Fecal pellets were collected at 3 and 6 months of age to analyze the gut microbiota by 16S rRNA gene sequencing. **a-b)** Differential effects of H_2_SO_4_-acidified and HCl-acidified drinking waters on the gut microbiota composition at the genus taxonomic level at 3 (a) and 6 months (b) of age. Columns and bars represent mean ± SEM and the circles show the individual data (n=5 mice, from 4-5 different cages for each group). Statistical significance was determined by 2-way ANOVA with Bonferroni’s post-test for multiple comparisons: *p<0.05, **^**p<0.01, ^&^p<0.001 ^#^p<0.0001; green lines and symbols: H_2_SO_4_-acidified vs. Non-acidified; red lines and symbols: HCl-acidified vs. Non-acidified; blue lines and symbols: H_2_SO_4_-acidified vs. HCl-acidified. Ph1, *Verrucomicrobia*; Ph2, *Tenericutes*; C1, *Clostridia*; C2, *Erysipelotrichia*; C3, *Verrucomicrobiae*; C4, *Mollicutes*; O1, *Clostridiales*; O2, *Erysipelotrichales*; O3, *Verrucomicrobiales*; O4, *Anaeroplasmatales*.

**Supplementary Fig. 7. Age-dependent changes in the genus composition of the gut microbiota in 129S6/SvEv male mice kept on non-acidified drinking water or received drinking water acidified with H_2_SO_4_ or HCl from weaning (postnatal day 21).** Fecal pellets were collected at 3 and 6 months of age to analyze the gut microbiota by 16S rRNA gene sequencing. Columns and bars represent mean ± SEM and the circles show the individual data (n=5 mice, from 4-5 different cages for each group). Statistical significance was determined by 2-way ANOVA with Bonferroni’s post-test for multiple comparisons: ^*^p<0.05, ^**^p<0.01, ^***^p<0.001, ^****^p<0.0001.
